# The Role of Microbiome and Probiotics in Chemo‐Radiotherapy‐Induced Diarrhea: A Narrative Review of the Current Evidence

**DOI:** 10.1002/cnr2.70029

**Published:** 2024-10-16

**Authors:** Sanaz Khorashadizadeh, Sara Abbasifar, Mohammad Yousefi, Farzad Fayedeh, AmirAli Moodi Ghalibaf

**Affiliations:** ^1^ Student Research Committee Birjand University of Medical Sciences Birjand Iran

**Keywords:** cancer, chemotherapy, diarrhea, probiotics, radiotherapy

## Abstract

**Background:**

In this article, we review the most recent research on probiotics effects on diarrhea in both human and animal models of the condition along with the therapeutic potential of these compounds based on their findings.

**Recent Findings:**

Nearly 50%–80% of cancer patients experience chemotherapy‐induced diarrhea (CID), serious gastrointestinal toxicity of chemotherapeutic and radiation regimens that leads to prolonged hospitalizations, cardiovascular problems, electrolyte imbalances, disruptions in cancer treatment, poor cancer prognosis, and death. CID is typically categorized as osmotic diarrhea. The depletion of colonic crypts and villi by radiotherapy and chemotherapy agents interferes with the absorptive function of the intestine, thereby decreasing the absorption of chloride and releasing water into the intestinal lumen. Probiotic supplements have been found to be able to reverse the intestinal damage caused by chemo‐radiation therapy by promoting the growth of crypt and villi and reducing inflammatory pathways. In addition, they support the modulation of immunological and angiogenesis responses in the gut as well as the metabolism of certain digestive enzymes by altering the gut microbiota.

**Conclusion:**

Beyond the benefits of probiotics, additional clinical research is required to clarify the most effective strain combinations and dosages for preventing chemotherapy and radiotherapy diarrhea.

**Abbreviations**
ALL: acute lymphocytic leukemia
CID: chemotherapy‐induced diarrhea
CTx: chemotherapy
DBI: diarrhea burden index
5‐FU: 5‐fluorouracil
GM: gut microbiota
IBD: inflammatory bowel disease
LrGG: *Lactobacillus rhamnosus* GG. probiotic
NGPs: new‐generation probiotics
RID: radiotherapy‐induced diarrhea
SCFAs: short‐chain fatty acids
WHO: World Health Organization

## 1 Introduction

Today, cancers become not only one of the most frequent chronic diseases worldwide but also a major cause of death [1]. According to the previous studies, cancer incidence is going to increase, as evidenced by the 23.6 million new cases of cancer diagnosed worldwide in 2019, the 10.0 million cancer deaths, and the estimated 250 million disability‐adjusted life years attributable to cancer; these statistics indicate raises of 26.3%, 20.9%, and 16.0%, respectively, since 2010 [2]. Among various therapeutic approaches for each cancer, chemotherapy and radiotherapy can be known as the most frequently used ones [3, 4]. In particular, radiation therapy, which will rob cancer cells of their capacity to proliferate, continues to be a crucial part of cancer treatment, with about 50% of all cases of cancer patients undergoing it at some time during their medical condition and accounting for 40% of cancer cures [3]. Despite the novel developments of medications in recent decades, chemotherapy continues to be the main treatment strategy for treating cancer [5]. Similar to radiotherapy, chemotherapy will prevent the growth of tumors and the proliferation of cancerous cells, which are typically caused by substances that interfere with the synthesis of DNA, RNA, or proteins in neoplastic cells or with the proper functioning of preformed molecules, thus avoiding invasion and metastasis [6]. In addition, the combination of both chemotherapy and radiotherapy is known as chemoradiotherapy, which is founded on the ideas of “spatial cooperation” and “in‐field cooperation,” with the ultimate aim of achieving synergistic antitumor effects from the collaboration of both treatment modalities [4, 7]. There are known side effects from chemotherapy, radiotherapy, and chemoradiotherapy in addition to their therapeutic effects. These side effects can range from mild gastrointestinal issues like nausea and vomiting to heart failure, pericardial effusion, pleural effusion, ischemic heart disease, and even death [8, 9]. Since 20%–45% of chemotherapy patients experience severe diarrhea during treatment, diarrhea is commonly referred to as the most concerning adverse event of chemotherapy. In fact, regimens involving capecitabine, 5‐fluorouracil (5‐FU), and irinotecan frequently cause diarrhea during chemotherapy [10, 11]. Due to the high prevalence of this side effect of chemoradiotherapy, several investigations have been conducted to find a way to prevent and treat chemoradiotherapy‐induced diarrhea; therefore, the current study aims to review the role that probiotics play in treating and preventing diarrhea caused by radiation, chemotherapy, and chemoradiotherapy.

## 2 Main Text

### 2.1 Microbiota and Its Role

Within the digestive tracts of both humans and animals, there exists a complex, diverse, and dynamic population of microorganisms known as the GM [12].

GM, comprised of various microorganisms such as viruses, bacteria, and some eukaryotes, establishes itself in the digestive tract post‐birth. As the body's primary micro‐ecosystem, the GM works in symbiosis with the host to maintain physiological functions in a state of dynamic equilibrium [13]. Key players in the GM, including Bacteroidetes, Firmicutes, and other anaerobic microorganisms, play a crucial role in converting dietary components into bioactive elements. For example, they break down indigestible carbohydrates like resistant starch, cellulose, and oligosaccharides into short‐chain fatty acids (SCFAs) like butyric, acetic, and propionic acids. These SCFAs have been found to possess anti‐inflammatory and chemotherapeutic properties, acting as tumor suppressors. Notably, patients with colon cancer show lower levels of butyrate‐producing bacteria.

Moreover, the GM contributes to the synthesis of essential vitamins such as K and B, bile acids, and cholesterol. It also plays a vital role in preventing local pathogen overgrowth, maintaining the integrity of the intestinal lining, and upholding immune system balance [14, 15, 16].

The formation of the GM can be affected by a host of circumstances and is highly dependent on the host and its surroundings. Dysbiosis is a disruption in the complex symbiosis between GM and the human body [17, 18]. The composition of the GM can be influenced by various factors, both intrinsic and extrinsic, including lifestyle, medications, diet, antibiotics, and diseases like inflammatory bowel disease (IBD) and obesity. To address disruptions in the symbiosis between the GM and the human body, therapeutic interventions like probiotics have been employed to restore the balance of the gut ecosystem [19, 20, 21].

#### 2.1.1 Gut Microbiota‐Mediated Diarrhea

The relationship between diarrhea and gut microbiota is currently gaining a lot of research. Diarrhea in people and animals is commonly associated with dysbiosis, a disorder caused by the dominance of pathogens like bacteria, fungi, and viruses. As pathogenic bacteria invade the digestive tract, they prevent normal bacteria from growing, which reduces the amount of good bacteria there [22]. Moreover, harmful compounds produced by pathogens lead to atypical immune responses and intestinal activity, which results in diarrhea. Here we discuss diarrhea caused by bacteria, fungi, and viruses [23].

##### 2.1.1.1 Bacteria and Diarrhea

Bacterial pathogen‐induced diarrhea is a worldwide health concern, particularly in developing nations. At the moment, the most common organisms associated with diarrhea are thought to be *Shigella*, *Salmonella*, *Campylobacter*, *Clostridium difficile* (*C. difficile*), and *Aeromonas*. In a study conducted on patients presenting to the emergency departments in Lebanon, it was found that 36% of stool samples contained *Campylobacter*, which makes it the most common cause of diarrhea incidents [24], whereas in another study, the majority of pathogens found in diarrheal stools (66.3% of cases) were enteroaggregative *Escherichia coli* (EAEC) [25]. *E. coli* is a facultative anaerobic bacterium that has a variety of diarrheal strains based on the physical manifestations, virulence processes, colonization sites, and pathological categories. These strains include *enterohemorrhagic E. coli*, *enteropathogenic E. coli*, *enteroaggregative E. coli*, *enteroinvasive E. coli*, and *enterotoxigenic E. coli* [26, 27].

Following infection, *E. coli* attaches itself to gastrointestinal epithelial cells via the adherent fimbriae, where it then generates toxins and has harmful consequences. Moreover, the majority of diarrheal *E. coli* show multidrug resistance, which makes it challenging to stop the infection from spreading [28]. *Salmonella*, the third most prevalent cause of diarrhea fatality, is classified as *nontyphoidal Salmonella* and *Salmonella typhi* based on its clinical manifestations [29].

The majority of *typhoid salmonella* infections, which result in 93.8 million foodborne illnesses and 155 000 fatalities annually, are found in underdeveloped nations. However, the individual's range of nontyphoid Salmonella is broad [30, 31].

*C. difficile* is a common bacterium found in both human and animal intestines that has the ability to produce spores [32]. Globally, *C. difficile* infection (CDI) is now the primary cause of antibiotic‐associated diarrhea. When the typical intestinal microorganism is disrupted, *C. difficile* colonizes and dominates in the large intestine, generating enterotoxin A and cytotoxin B [33]. These toxins cause significant inflammation of the intestines, diarrhea, and pseudomembranous colitis by further damaging the cytoskeleton of epithelial cells [34]. Moreover, by altering the local microbial population, *C. difficile* may be able to manufacture indole, which will have an impact on the quantity and variety of bacterial communities in the colon [35]. *Shigella* is a type of gram‐negative bacteria that can cause diarrhea in both humans and animals. An estimated 125 million cases of diarrhea and 160 000 fatalities are attributed to *Shigella* each year, with young children accounting for a third of these cases. *Shigella* invades the intestinal cavity and releases *Shigella* enterotoxin and serotype toxin 1, which further penetrate and kill the large intestine's epithelial cells and ultimately cause severe diarrhea that is mucus‐like, bloody, or watery [36].

##### 2.1.1.2 Fungus and Diarrhea

A significant component of gut microbes are fungi, and it has been established that specific fungal communities are strongly linked to diarrhea [37]. Although its exact mode of action is yet unknown, candida is typically recognized as a trustworthy source of diarrhea [38]. In a clinical context, it is generally accepted that this fungus may induce diarrhea only in certain cases. *Candida albicans* (*C. albicans*) is the most prevalent fungus in the mammal's intestinal tract. It has long been debatable how *C. albicans* and diarrhea are related. However, in a mouse model, it was discovered that this yeast may cause intestinal dysbiosis [39].

##### 2.1.1.3 Virus and Diarrhea

The viral microbiome is a complicated combination of DNA viruses, eukaryotic RNA viruses, and bacteriophages, which preserves both human and animal health. According to earlier research, some viruses can cause diarrhea in both humans and animals. Viral diarrhea manifests as fever, diarrhea, and vomiting [40]. According to a systematic analysis of diarrheal burdens in 195 countries in 2016, *Rotavirus* was the most common cause of diarrheal incidence and mortality in children under the age of five (128 515 deaths, 105 138–155 133) and all age groups (228 047 deaths, 183 526–292 737) [41]. When intestinal epithelial cells are infected by *Rotavirus*, it stimulates gastrointestinal secretion and activates the nervous system in the intestines, which destroys absorbent intestinal epithelium and kills intestinal cells which results in diarrhea [42].

#### 2.1.2 Strategies to Mitigate Gut Microbiota

Prebiotics are “selectively fermented substances that allow particular modifications, both in composition and/or activity in the gut microbiota that provide benefits upon host wellness and health” [43]. A prebiotic's major properties include resistance to the action of digestive enzymes in the gut, fermentability by colonic bacteria, and bifidogenic and pH‐lowering actions. Prebiotics suppress certain strains of possibly dangerous bacteria, particularly Clostridium, hence preventing diarrhea [44]. In a mouse model of colitis, prebiotic therapy significantly decreased intestinal inflammation [45]. Also, in another study, prebiotic alleviated the severity of dextran sodium sulfate colitis by altering the intracolonic environment [46].

Regardless of encouraging animal research, there has been no evidence of effective preventive or therapeutic usage of prebiotics in individuals with diarrhea and/or gastrointestinal inflammation. This could be due to side effects like gas, borborygmus, discomfort, or diarrhea, which can occur when therapeutic doses of prebiotics are supplied to highly sensitive people, irritable bowel syndrome (IBS) patients, or those with a maladapted gut flora [47, 48].

Fecal microbiota transplantation (FMT) is an approach that recovers dysbiosis by transplanting stool from a healthy donor to the patient [49]. A number of studies suggest that restoring a healthy microbe population is an effective way to prevent or treat gastrointestinal disorders [50, 51]. In particular, FMT is an extremely effective treatment for CDI, IBD, and IBS [52, 53, 54]. In an FMT trial, it was suggested that FMT can alleviate diarrhea in pre‐weaning calves with gut microbiota changes and that FMT may have a role in improving growth performance [55]. Additional studies with bigger cohorts will be required to validate this and find the best FMT method.

### 2.2 Probiotics

Probiotics are live supplementary microorganisms such as *Lactobacillus* or *Bifidobacterium* that are beneficial to the human body by restoring the host's gut flora when taken in adequate doses [56, 57]. The World Health Organization (WHO) defines probiotics as alive, viable, and nonpathogenic bacteria; they do not comprise fragments of decomposed microorganisms. In addition, to have the greatest impact, probiotics must be resistant to gastrointestinal conditions such as low pH and maintain their ability to be adhered and colonized to the intestinal tract [58]. Many genera and phylum of fungi and bacteria are shown to possess probiotic qualities; however, the *Lactobacillus* and *Bifidobacterium* species are most employed as probiotics. Other bacterial genera, including *Streptococcus*, *Enterococcus*, and *Bacillus*, in addition to species of the yeast genus *Saccharomyces*, can also exhibit probiotic qualities (Table 1) [59, 60]. It should be noted that not all strains of a genus have probiotics characteristics. The safety of probiotics depends on the strain's origin, antibiotic resistance, and inability to pathogenicity [61, 62].

**TABLE 1:** Common species of probiotic microorganisms.

Lactobacilli	Bifidobacterium	Other bacteria	Non‐bacterial
*Lactobacillus acidophilus* *Lactobacillus johnsonii* *Lactobacillus gasseri* *Lactobacillus casei* *Lactobacillus rhamnosus* *Lactobacillus plantarum* *Lactobacillus bulgaricus*	*Bifidobacterium longum* *Bifidobacterium breve* *Bifidobacterium bifidum* *Bifidobacterium infantis*	*Streptococcus thermophilus* *Enterococcus faecium* *Bacillus coagulans*	*Saccharomyces cerevisiae* *Metschnikowia* *Hanseniaspora* *Candida* *Kluyveromyces* *Pichia* *Debaryomyces*

Probiotics can adjust the gut microbiota's population and maintain a healthy bacteria balance within the gastrointestinal tract. Their function has not been determined, although many theories have been put forth; probiotic bacteria in the gut produce SCFAs such as butyrate, acetate, and propionate, which are antibacterial substances that maintain a healthy gut environment. They can also diminish intraluminal pathogenic bacteria by generating competitive inhibition, decreasing the permeability of epithelia, and lowering luminal pH. Moreover, these microorganisms improve the gut microbiota and promote lactase synthesis, which aids in the digestion of lactose [63]. Probiotics are now widely recognized as having positive effects on different digestive conditions including necrotizing enterocolitis, acute infectious diarrhea, and antibiotic‐induced diarrhea, and many new probiotic‐containing products and food have been developed [64, 65]. Studies on human and animal subjects with IBD have demonstrated that the application of probiotic organisms can successfully reduce intestinal inflammation by changing the composition, metabolism, and functional characteristics of intestinal flora [66]. Furthermore, *Bacillus coagulans* among other probiotics species showed to be effective in ameliorating IBS symptoms such as bloating, straining, and abdominal pain [67]. In another study, patients with mild to severe ulcerative colitis achieved remission after consumption of probiotic cocktail VSL#3 [68]. In addition, it was found that consuming probiotics reduces the symptoms of gastrointestinal reflux disease including heartburn and regurgitation [69].

The crucial role of commensal bacteria in the development of cancer has been demonstrated by increasing data. Recent research has concentrated on targeting microbiota to prevent cancers and improve therapeutic effectiveness. It was discovered in a study that the administration of soy isoflavones and *Lactobacillus casei Shirota* was linked to a lower rate of breast cancer [70]. Moreover, taking *Streptococcus thermophilus* and *Lactobacillus delbrueckii* decreased the risk of developing colorectal cancer (CRC) [71]. Probiotics also benefit by reducing treatment toxicity, postoperative complications, and poor quality of life. Various studies indicated that taking probiotics before or during chemotherapy effectively prevents combined immunodeficiency. They were also effective in prevention of diarrhea in pelvic cancer patients undergoing radiotherapy (RTx). Herein, we reviewed some studies regarding the efficacy and safety profile of using probiotics for the treatment of radiation‐ or chemotherapy‐induced diarrhea.

#### 2.2.1 Probiotics' Function in Preserving the Gut Barrier

Various research studies have established that probiotics can improve the signaling pathway that is responsible for assembling tight junction complexes, which are crucial for maintaining the integrity of the epithelial barrier. For example, *Lactobacillus plantarum* (*L. plantarum*) has been demonstrated to enhance the physical barrier of the intestinal wall by increasing the production of certain proteins, including zonula occludens‐1 (ZO‐1), zonula occludens‐2 (ZO‐2), and occludin, while decreasing the production of claudin‐1 and claudin‐2. In addition, research has revealed that probiotics can reduce the levels of inflammatory cytokines, such as IL‐6, IL‐1β, TNF‐α, myeloperoxidase (MPO), interferon‐γ (IFN‐γ), and p65, myosin light chain 2 (MLC2), and myosin light chain kinase (MLCK). They also elevate the levels of anti‐inflammatory cytokines, including IL‐10, transforming growth factor‐β1 (TGF‐β1), and TGF‐β2, which hinder the NF‐κB signaling pathway. These results suggest that probiotics have protective effects against inflammation [72, 73, 74].

Cancer patients receiving chemotherapy are known to experience gut dysbiosis. It was discovered that several taxa, including *Ruminococcus*, *Lachnospira*, *Roseburia*, *Faecalibacterium*, and *Clostridium*, were reduced following chemotherapy [75]. These taxa are shown to minimize inflammation by modulating the NF‐ƙB pathway [76]. *Bifidobacterium*, which is reduced after chemotherapy, also is capable to prevent inflammation in intestinal epithelium by reducing the inflammatory response brought on by lipopolysaccharide and TNF‐α [77]. Moreover, numerous taxa that get depleted after chemoradiotherapy such as *Roseburia*, *Ruminococcus*, *Bifidobacterium*, *Coprococcus*, and *Faecalibacterium* are butyrate‐producing bacteria, leading to decreased formation of SCFAs, which are known to maintain colonic mucosal homeostasis and restrict inflammatory response [78]. In several studies, the number of *Enterobacteriaceae* increased following treatment [75, 79]. In addition, a rise in the metabolisms of nitrogen and sulfur was observed, confirming the connection between oxidative stress and an increase in *Enterobacteriaceae* [75].

**FIGURE 1:**
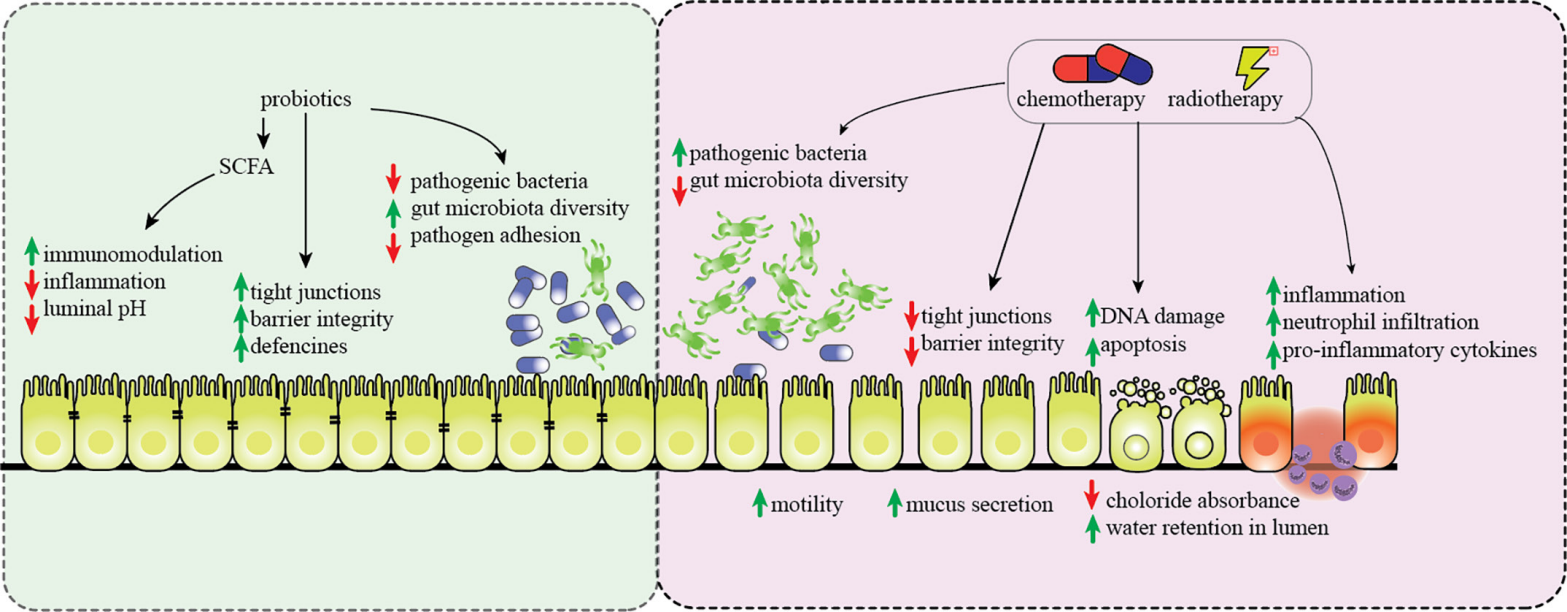
A summary of the probiotic's effects on chemotherapy‐ and radiotherapy‐induced diarrhea. Chemo‐ and radiotherapy increase the pathogenic bacteria, increase mucus secretion and water retention, increase inflammation and apoptosis, and result in reduced barrier integrity. Probiotics, on the other hand, reduce inflammation, increase barrier integrity and defenses, and tend to reduce pathogenic bacteria and their adhesion to gut.

Probiotics can stimulate cytoprotective pathways within epithelium, block reactive oxygen species and free oxygen radicals, interact with cellular junctions, and initiate the NF‐*κ*B signaling pathway to improve the integrity of the mucous and provide the establishment of a native immune reactions [78, 80, 81]. As a result, it helps to maintain barrier function, regulate intestinal homeostasis, protect the gut from damage, promote tissue regeneration, and support healthy intestinal microbiota; however, several evidences suggested further required studies to indicate their effects (Figure 1).

#### 2.2.2 Synbiotics and Their Benefits

According to the WHO, prebiotics are indigestible food components that have a positive impact on the host's well‐being by promoting the growth and activity of beneficial microorganisms [82]. Prebiotics facilitate the growth and function of probiotics such as *Bifidobacterium* and *Lactobacillus* by acting as specific substrates for them [83]. SCFAs like acetate, propionate, and butyrate are produced when gut microorganisms ferment prebiotics [84]. SCFAs regulate host metabolism and appetite regulation, enhance the function of the intestinal barrier, and have anti‐inflammatory properties [85, 86]. In addition, prebiotics can directly influence host immune cells and receptors to alter immune responses. Modifications in the gut microbiota caused by prebiotics also have an indirect impact on mucosal and systemic immunity [87]. Prebiotics have also been demonstrated to enhance metabolic health indicators, such as lowering insulin resistance, dyslipidemia, and obesity. These metabolic advantages are facilitated by prebiotics' bifidogenic and SCFA‐producing properties [87, 88].

The potential advantages of combining prebiotics and probiotics to create “synbiotics” have been discussed. According to research, this synergistic combination can enhance the development and survival of commensal microbes in the digestive system [89]. According to research, taking supplements containing synbiotics can boost the number of *Bifidobacterium* and *Lactobacillus* species—two types of good gut bacteria—in the body [90]. This may result in better digestion, a lower chance of diarrhea, and alleviation from ailments like IBS [91]. Synbiotics have shown promise for improving metabolic health in addition to the gut. According to certain studies, synbiotic therapies can help type 2 diabetics with their insulin sensitivity and glucose control markers. By modifying the gut microbiome and lowering inflammation, synbiotics may also aid in the management of nonalcoholic fatty liver disease [83]. The possibility of using synbiotics to improve immunological function has also been studied. According to some research, synbiotic formulations can modify inflammatory pathways and raise levels of good gut antibodies [92].

The purpose of synbiotics is to solve potential probiotic survival issues. The use of synbiotics seems to be justified by data demonstrating increased probiotic bacterial survival throughout upper gastrointestinal tract transit [93]. In synbiotic compositions, the main prebiotics is oligosaccharides like fructooligosaccharide (FOS), xylooligosaccharides (XOS), and GOS, as well as those derived from natural sources such as chicory and yacon roots, while probiotic strains used in these compositions include *Lactobacilli, Bifidobacteria* spp., *Saccharomyces boulardii*, and *B. coagulans*. Synbiotic intake seems to provide the following health benefits: (1) a modulated gut microbiome with higher concentrations of lactobacilli and bifidobacteria, (2) enhanced immunomodulatory capability, and (3) preventing bacterial translocation [94].

### 2.3 Pathogenesis of Chemoradiotherapy‐Induced Diarrhea

#### 2.3.1 Chemotherapy‐Induced Diarrhea

Cellular damage of the intestine is apparent almost immediately after chemotherapy (CTx) is started. Abdominal pain, nausea, vomiting, constipation, and diarrhea are a few symptoms connected to CTx. Direct injury to the mucosa is the main source of these symptoms [95]. Chemotherapy‐induced diarrhea (CID) can cause dehydration, malnutrition, and hospitalization. These complications can cause impairments within the cardiovasculature and increase rates of mortality. In addition, diarrhea may hinder and interfere with cancer treatment by resulting in dosage reductions or delays, which may have an effect on survival. Irinotecan (CPT‐11), capecitabine, and 5‐FU are among the leading therapeutics that frequently lead to diarrhea [96, 97]. Acute intestinal mucosal injury, such as the loss of intestinal epithelial cells, superficial necrosis, and inflamed gut, appears to be responsible for CID, which leads to a discrepancy between absorbing and secreting in the small intestine.

##### 2.3.1.1 Irinotecan‐Induced Diarrhea

Irinotecan is frequently administered as therapy of metastatic CRC in first‐ and second‐line treatment. Regardless of when it is administered, the most frequent adverse effects of irinotecan are myelosuppression and delayed‐type diarrhea [98]. Irinotecan can result in either acute diarrhea that occurs shortly after receiving the medication or delayed diarrhea. Acute cholinergic properties produce immediate‐onset diarrhea, which is frequently accompanied by additional signs of cholinergic excess such as abdominal cramps, lacrimation, rhinitis, and salivation [99]. The average symptom duration is 30 min, and atropine usually has a quick effect. When diarrhea develops more than 24 h following irinotecan delivery, it is referred to as delayed‐type diarrhea. This condition can happen at any dose level and is noncumulative [100]. Active metabolite 7‐ethyl‐10‐hydroxycamptothecin (SN38) is produced by metabolization of irinotecan via hepatic and peripheral carboxylesterase, which is then glucuronidated by hepatic uridine diphosphate glucuronosyltransferase‐1A1 (UDP‐GT 1A1) to SN38‐glucuronide (SN38G). SN‐38 and SN‐38G are both eliminated through the bile and urine. Within the gut, bacterial β‐glucuronidase deconjugates SN38G to SN38. The presence of more SN38 in feces than SN38G suggests that there is a significant β‐glucuronidase activity in the intestinal contents. Irinotecan‐induced diarrhea is caused by the free intestinal SN38, either from deconjugation of SN38G or bile. There is considerable disagreement regarding the exact processes by which the free SN38 causes diarrhea [101].

##### 2.3.1.2 Fluoropyrimidines (5‐FU, Capecitabine, Tegafur/Uracil)

Fluoropyrimidines have been linked to severe diarrhea, which can be deteriorated by including leucovorin (LV) in the therapy regimen. The severity of diarrhea could also worsen when 5‐FU is given intravenously rather than as a bolus injection. Diabetes, female gender, and Caucasian race are some clinical risk factors for fluoropyrimidine‐induced diarrhea [102]. Even though 5‐Fu is commonly utilized in malignancy therapies and is associated with causing diarrhea, very few fundamental studies have made an effort to unravel the processes behind its pathophysiology. However, prior research identified that the depletion of colonic crypts by fluoropyrimidines interferes with the absorptive function of the intestine, thereby lowering the absorption of chloride and resulting in discharge of water into the intestinal lumen [103].

#### 2.3.2 Radiation‐Induced Diarrhea

The pathophysiology of radiotherapy‐induced diarrhea (RID) includes different mechanisms. First of all, the intestine has a sophisticated microbial environment that serves a crucial and distinct purpose. Since radiation regimens nonspecifically attack rapidly dividing cells by targeting biological DNA as well as proteins, lipids, carbohydrates, and other compounds, they can change the composition of the intestinal microbiota and host homeostasis which might cause the mitotic arrest of the mucosal epithelium [104]. Secondly, the alteration of connective tissues results in vascular injuries, which, when severe, cause fibrosis, stenosis, and a decrease in gastrointestinal movement. This, as a result, makes the mucosal cells more permeable, encouraging the transition of bacteria from the digestive tract to the mucosa which interferes with the host's immune responses. Inflammation then quickens the radiation response and expresses more of the endothelial cell adhesion molecules which cause leukocytes to adhere to the vessels of endothelium and lead to inflammatory cells extravasating to the inflammatory tissue [105]. Moreover, radiation can stimulate the central nervous system and increase gastrointestinal movement. It can also reduce bile acid reabsorption by depleting villi and decreasing the absorptive surface area of the small intestine [104, 106].

### 2.4 Probiotics and Chemoradiotherapy‐Induced Diarrhea

Possible advantages of probiotics both during and after chemotherapy or radiotherapy to enhance cancer treatment safety and reduce chemo radio‐gastrointestinal side effects have been investigated previously. The main causes of gastrointestinal toxicity during chemotherapy and radiotherapy are mucosal injury, reduced colonization resistance, and shifts in the host's gut microbiota. Probiotics can decrease the likelihood and severeness of CTx or RTx treatment side effects including diarrhea [107]. Here, we are going to review the current knowledge around the effects of probiotics on chemoradiotherapy‐induced diarrhea in both animal models and human studies. Promising outcomes in animal models (Table 2) and clinical studies (Tables 3 and 4) have been reported. Preclinical and clinical studies showed that administration of probiotics prior to, during, and following CTx or RTx resulted in reduced episodes of severe diarrhea and demonstrated the safety of utilizing probiotic products despite using different study designs, animal populations, and probiotic brands. The employed probiotic was the only comparable component between animal and clinical investigations; therefore, we listed the study strains in Table 5.

**TABLE 2:** Research on probiotics to cure and prevent radiation and chemotherapy.

Author	Main objective	Cases/Controls	CTx or RTx regimen	Type of Probiotics	Results
Zhu et al. [120]	To look at *Se‐B. longum* DD98's potential defences against hepatic and intestinal Harmful effects brought on by CPT‐11	Six groups of eight male BALB/c mice, each aged 5–7 weeks, were randomly assigned: Control CPT‐11 alone CPT‐11 + Na_2_SeO_3_ CPT‐11 + B. longum DD98 CPT‐11 + low dosage of *Se‐B. longum* DD98 (0.2 mg Se/kg) CPT‐11+ high dosage of *Se‐B. longum* DD98 (0.05 mg Se/kg)	CPT‐11 injections intraperitoneally at a dose of 75 mg/kg for 4 days in a row	For 21 days prior to the therapy and 7 days following treatment, two different dosages of *Se‐B. longum* DD98 were given: 0.05 mg Se/kg and 0.2 mg Se/kg, respectively	Six days following the initial CPT‐11 injection, the degree of diarrhea peaked, with 87.5% of mice experiencing severe diarrhea. Treatment with Na, BL, SeL, and SeH reduced the intensity of diarrhea to varying degrees; by the seventh day, the rates of severe diarrhea were reduced to 50%, 75%, 50%, and 25%, respectively.
Gigola et al. [108]	To assess the effect of probiotics on rats treated with capecitabine for colorectal cancer in terms of overall survival and quality of life	50 Male Wistar–Lewis rats, aged 8 weeks, randomly divided into one of the five groups listed below: Control Control + probiotic 1,2‐DMH alone 1,2‐DMH + capecitabine 1,2‐DMH + probiotic 1,2‐DMH + capecitabine + probiotic	An oral gavage of capecitabine was given for period of 14 days, with 7 days off, at a median toxic dosage (359 mg/kg)	Oral administration of 1 mL of the probiotic mixture containing *Streptococcus faecalis*, *L. plantarum*, *L. casei*, and *Bifidobacterium brevis* was started 7 days prior to the initiation of carcinogenesis induction and continued cyclically (5 consecutive days followed by a 5‐day break) until the animal's death	Compared to the capecitabine group, the probiotic and capecitabine group had a significantly greater overall survival (*p* = 0.001). By the time of death, the capecitabine and probiotic group gained more body weight than the capecitabine alone did (*p* = 0.0001). Neither capecitabine regimens nor the main tumor were found to cause diarrhea or any other symptoms in rats receiving probiotic supplements.
Huang et al. [110]	Initially, to study probiotic safety; subsequently, to assess their effects on 5‐Fluorouracil intestinal mucositis	Thirty‐six male SCID/NOD mice were randomly assigned to six groups: Control Probiotic suspension of Lcr35 Probiotic suspension of LaBi 5‐FU + saline 5‐FU + probiotic suspension of Lcr35 5‐FU + probiotic suspension of LaBi	Intraperitoneal (IP) injection of a single dosage of 30‐mg/kg/day 5‐FU was given for 5 days	For 5 days, a daily dose of 100 μL of a probiotic containing Lcr35 (*L. casei* variety rhamnosus) or LaBi (*L. acidophilus*, and *B. bifidum*) was administered	Day 5 diarrhea scores were considerably reduced in both probiotic groups (1.33 ± 0.17 (*p* < 0.05) for Lcr35 and 1.42 ± 0.24 (*p* < 0.05) for LaBi) compared to 5‐FU alone. More than 10% of the body weight was decreased by the 5‐FU group without probiotics, but all groups saw weight loss when compared to the control group. Comparing Lcr35 to LaBi, the protective effect was significantly (*p* < 0.05) stronger. The administration of 100 μL of the probiotics *L. casei* variety rhamnosus (Lcr35) or *L. acidophilus* and *B. bifidum* (LaBi) restored crypt depth to normal levels (*p* = 0.05), However, it had no significant influence on height.
Souza et al. [114]	To evaluate the protective effects of *A. muciniphila* BAA‐835 against mucositis in mice modeled by chemotherapy	To assess the impact of dosage response; 42 conventional female BALB/c mice aged 6–8 weeks were split up into 6 groups, with 7 mice in each group: Control Probiotic ($10^{10}$ CFU/mL BAA‐835) Mucositis (5‐fluorouracil) Treatment group ($10^{8}$ CFU/mL BAA‐835 + 5‐FU) Treatment group ($10^{9}$ CFU/mL BAA‐835 + 5‐FU) Treatment group ($10^{10}$ CFU/mL BAA‐835 + 5‐FU) To evaluate the viability (living or heat‐killed): 35 conventional female BALB/c mice aged 6–8 weeks, split up into five groups (*n* = 7): Control Probiotic ($10^{10}$ CFU/mL BAA‐835) Mucositis (5‐fluorouracil) Treatment group (viable $10^{10}$ CFU/mL BAA‐835 + 5‐FU) Treatment group (inactivated $10^{10}$ CFU/mL BAA‐835 + 5‐FU) To evaluate the effects of treatment procedures: 35 conventional female BALB/c mice aged 6–8 weeks, split up into five groups (*n* = 7): Control Probiotic ($10^{10}$ CFU/mL BAA‐835) Mucositis (5‐fluorouracil) Preventive treatment group (viable $10^{10}$ CFU/mL BAA‐835 + 5‐FU) Curative treatment group (viable $10^{10}$ CFU/mL BAA‐835 + 5‐FU)	5‐fluorouracil was administered intraperitoneally at a single dose of 300 mg/kg	0.1 mL of an intragastrically administered solution containing different concentrations ($10^{8}$ CFU/mL, $10^{9}$ CFU/mL, or $10^{10}$ CFU/mL) of *A. muciniphila* BAA‐835 was given for a duration of 14 days	When given at a dosage of $10^{10}$ CFU/mL, *A. muciniphila* BAA‐835 significantly reduced both weight loss (*p* = 0.01) and small intestine shortening (*p* = 0.002) in rats following chemotherapy. *A. muciniphila* BAA‐835 treatment led to a significant reduction in symptoms, which lessened the intensity of the condition and helped the BAA‐835 + 5‐FU group's DAI evolution on days 2 and 3 (*p* = 0.001, and *p* = 0.006, respectively) following disease induction to evolve more slowly than 5‐FU group.
Tooley et al. [118]	To assess the TH‐4's effect on the development of MTX‐induced small intestine mucositis in tumor‐bearing rats	36 Female dark agouti tumor‐bearing (mammary adenocarcinoma) rats were divided into the following groups: Saline control MTX alone TH‐4 alone MTX + TH‐4	At 0 and 24 h, 1.5 mg/kg intramuscular methotrexate (MTX) was given	Oral *Streptococcus thermophilus* was given between −48 and +96 h after MTX	MTX‐administered rats lost weight more significant than saline or TH‐4 animals (*p* < 0.001). Semiquantitative small intestine damage scores showed that regardless of TH‐4 treatment, all rats given MTX had severely injured jejunal and ileal mucosa in comparison to saline and TH‐4 controls (*p* = 0.05). There were no changes between the MTX controls and the animals treated with MTX + TH‐4. The proximal jejunum sustained the most damage
Wang et al. [117]	To study how *S. thermophilus* TH‐4 affects doxorubicin‐induced mucositis	32 Female dark agouti rats were randomly assigned into four groups (*n* = 8 rats/group): Milk + saline Milk + doxorubicin TH‐4 + saline TH‐4+ doxorubicin	On Day 6, rats were intraperitoneally injected with 0.5 mL of a single dose of doxorubicin (20 mg/kg)	*S. thermophilus* TH‐4 ($10^{10}$ CFU/mL) was given daily via orogastric gavage for 9 days	In the present work, oral *S. thermophilus* TH‐4 administration (109 CFU/mL) had a negligible effect on the clinical and biochemical insults brought on by doxorubicin‐induced mucositis in the rat.
Yuan et al. [115]	To see if *B. infantis* could treat rats with intestinal mucositis brought on by 5‐FU	Thirty male Sprague–Dawley rats, split up into three groups: 1. Control (*n* = 10) 2. 5‐FU (*n* = 10) 3. 5‐FU + *B. infantis* (*n* = 10)	On Day 7, an intraperitoneal injection of 150 mg/kg body weight of 5‐FU was administered as a single dosage	*B. infantis* (1× $10^{9}$ CFU) was given orally by gavage for 11 days, beginning 7 days before to the 5‐FU injection	Rats given *B. infantis* showed a partial prevention of body weight loss (*p* < 0.05, 5‐FU + *B. infantis* group vs. 5‐FU group). Before receiving the 5‐FU injection, *B. infantis* medication did not cause any diarrhea. It also lessened the amount of diarrhea that the rats experienced from the 5‐FU injection, ranging from normal stool excretion to mild diarrhea (*p* < 0.05, 5‐FU + *B. infantis* group vs. 5‐FU group).
Yeung et al. [109]	To examine the possible impacts of probiotics on chemotherapy‐induced mucositis	72 male Balb/c mice (*n* = 12 mice/group): Control Probiotic suspension of Lcr35 Probiotic suspension of LaBi 5‐FU + saline 5‐FU + probiotic suspension of Lcr35 5‐FU + probiotic suspension of LaBi	30 mg/kg/day 5‐FU was given intraperitoneally for 5 days	For a period of 5 days, 100 μL of probiotics comprising $10^{7}$ CFU, either *L. acidophilus* and *B. bifidum* (LaBi) or *L. casei* variety rhamnosus (Lcr35), were given every day	The 5‐FU groups had significant diarrhea; however, diarrhea was reduced and the diarrhea score significantly improved following treatment with Lcr35 and LaBi.
Yuan et al. [112]	To look into how probiotics affect gut flora and whether they can help with diarrhea brought on by chemotherapy Probiotic consists of 10 mg of *Clostridium butyricum* TO‐A 1× 105–1× 108, 10 mg of *Bacillus mesentericus* TO‐A: 1× 105–1× 108, and 2 mg of *Streptococcus faecalis* T‐110: 2× 105–4× 108	48 Kunming female mice randomly divided into four groups (*n* = 12): Control Oxaliplatin BIO‐THREE probiotics Oxaliplatin BIO‐THREE probiotics	30 mg/kg oxaliplatin was administered intraperitoneally twice on Days 0 and 6	A daily gavage of 0.5 mL of the probiotic cocktail BIO‐THREE (200 mg tablet) was administered. Each tablet has 10 mg of *Bacillus*, 10 mg of *Clostridium butyricum*, and 2 mg of *Streptococcus faecalis*	Oxaliplatin‐treated mice showed significant alterations in their daily food and water intake, which was associated with their decrease of bodyweight. Mice intestine showed significant elevation in TNF‐α, suggesting that oxaliplatin can disrupt the digestive system and damage the intestines. Probiotic‐therapy decreased intestinal TNF‐α, although it had a few negative impacts on the mice's liver, diet or growth. Thus, consuming probiotics might help in minimizing the digestive side effects brought on by oxaliplatin.

Abbreviations: 1,2‐DMH, 1,2‐dimethylhidrazine dihydrochloride; 5‐FU, 5‐flurouracil; CFU, colony forming unit; CPT‐11, irinotecan; LaBi, *Lactobacillus acidophilus* and *Bifidobacterium bifidum*; Lcr, *Lactobacillus rhamnosus*; MTX, methotrexate; Se‐B, selenium‐enriched Bifidobacterium; TNF, tumor necrosis factor.

**TABLE 3:** Investigations on the clinical benefits of probiotic supplementation in the management and avoidance of radiation‐induced diarrhea.

Author	Main objective	Case/Control	Type of probiotics	Cancer	CTx or RTx regimen	Result
Du et al. [122]	To evaluate the protective effects of *Bacillus licheniformis* preparation on gastrointestinal disorders and inflammation induced by radiotherapy in pediatric with central nervous system tumor	Experiment group (*n* = 80) Control group (*n* = 80)	ZCS	Central nervous system tumor (glioblastoma [*n* = 48], medulloblastoma [*n* = 60], ependymocytoma [*n* = 34], and astrocytoma [*n* = 18]	36 Gy of craniospinal irradiation and 1.5 Gy of posterior fossa boost	It has been shown that ZCS significantly reduced gastrointestinal toxicity, including diarrhea.
Demers et al. [121]	To assess the impact of probiotics on diarrhea in individuals treated with pelvic radiotherapy	Placebo (*n* = 29) Standard dose of Bifilact (*n* = 32) High dose of Bifilact (*n* = 18)	Bifilact: *Lactobacillus acidophilus* LAC‐361 + *Bifidobacterium longum* BB‐536	Malignancies of the prostate, gynecologic cancers without chemotherapy, and gynecologic or rectal cancers with chemotherapy	Treatments with radiation therapy for at least 40 Gy at the pelvic level, either with or without chemotherapy	Overall, there was no discernible difference in Grade 2–3–4 diarrhea between the groups (*p* = 0.13). At 60 days, the hazard ratio was 0.69 (*p* = 0.04), meaning that more patients in the standard dose group (35%) than in the placebo group (17%) did not have moderate to severe diarrhea. Compared to the placebo group, the postoperative patients in the normal probiotic dose group had a greater percentage of patients without extremely severe diarrhea (97% versus 74%; *p* = 0.03).
Ahrén et al. [10]	Assessing the potential of probiotics to reduce the side effects of pelvic radiation therapy	High dose probiotic (*n* = 34) Low dose probiotis (*n* = 32) Placebo (*n* = 31)	*Lactiplantibacillus plantarum* HEAL9 combined with *Lactiplantibacillus plantarum* 299	Gynecologic cancer	Treatment with external beam radiation to the pelvis, with a minimum dose of 40 Gy	There was no statistically significant decrease in the mean quantity of loose stools (Bristol stool types 6 and 7) among the probiotic groups. On the other hand, the high‐dose probiotics group experienced 8.65 ± 5.93 days with at least one loose stool, compared to 15.04 ± 8.92 days in the placebo group (*p* = 0.014).
Thursby et al. [12]	To evaluate the impact of probiotis on radiation‐induced diarrhea with or without honey	Probioc (n = 22) Probioc‐ Honey (*n* = 21) Placebo (*n* = 24)	LactoCareO: *Lactobacillus casei* *Lactobacillus acidophilus* *Lactobacillus rhamnosus* *Lactobacillus bulgaricus* *Bifidobacterium breve* *Bifidobacterium longum* *Streptococcus thermophilus*	Malignancies of the pelvis including colorectal, endometer, prostate, ovary, bladder, cervix, bone, and sarcoma	Radiation with 18 MV at a cumulative dosage of between 4000 and 5000 cGy (1.8 Gy/day)	The results showed that patients who took probiotics or probiotic plus honey had decreased daily bowel movements (*p* = 0.003 and 0.006), diarrhea grade (*p* = 0.001 and 0.001), the need for anti‐diarrheal medications (*p* = 0.021 and 0.041), and increased stool consistency (*p* = 0.004 and 0.005).)

**TABLE 4:** Investigations on the clinical benefits of probiotic supplementation in the management and avoidance of chemotherapy‐induced diarrhea.

Author	Main objective	Case/Control	Type of probiotics	Cancer	CTx or RTx regimen	Result
Lacouture et al. [126]	To assess preventive care for advanced non‐small cell lung cancer patients taking 45 mg of dacomitinib orally on a continuous basis to prevent gastrointestinal and dermatological side effects	*Cohort 1*: 1. Prophylactic treatment of Doxycycline (*n* = 56) 2. Placebo (*n* = 58) *Cohort 2*: Prophylactic treatment of alclometasone diproprionate cream and VSL#3 probiotic	VSL#3 probiotic	Non‐small‐cell lung cancer	Dacomitinib 45 mg orally on a continuous basis	The frequency of either all‐causality, all‐grade diarrhea or all‐causality, grade ≥ 2 diarrhea was unaffected by the VSL#3 probiotic when compared to placebo or doxycycline. The frequency of grade II diarrhea was lower with doxycycline than with placebo, although the difference was not statistically significant.
Eghbali et al. [127]	To assess how LactoCare synbiotic treatment affects children with ALL who are undergoing chemotherapy‐induced nausea, vomiting, diarrhea, and constipation	Synbiotic group *n* = 54 Placebo group *n* = 52	LactoCare capsules: *Lactobacillus casei* *Lactobacillus acidophilus* *Lactobacillus rhamnosus* *Lactobacillus bulgaricus* *Bifidobacterium breve* *Bifidobacterium longum* *Streptococcus thermophilus* Prebiotic fructooligosaccharides	Acute lymphoblastic leukemia (ALL)		In the LactoCare therapy group, CID was observed in 3.7% and 1.8% of patients on the first and second days, respectively, but not at all on the third through seventh days (*p* < 0.05). Less than 10% of CID instances occurred in the placebo group on the first, fifth, sixth, and seventh days, but 11.5%, 13.5%, and 11.5% of cases occurred on the second, third, and fourth days, respectively.
Wada et al. [130]	To assess the impact of *Bifidobacterium breve* administered enterally on children receiving chemotherapy for childhood cancers	Case (probiotic): *n* = 19 Control (placebo) l: 23	*Bifidobacterium breve* strain Yakult BBG‐01	Cases: Acute lymphoblastic leukemia [6] Non‐Hodgkin lymphoma [6] Yolk sac tumor [4] Ewing sarcoma [2] Control: Acute lymphoblastic leukemia [11] Acute myeloid leukemia [2] Non‐Hodgkin lymphoma [4] Huntington's disease [2] Primitive neuroectodermal tumors [2] Leiomyosarcoma [1]		The frequency and length of diarrhea were not significantly different across groups (*p* values = 0.23 and 0.09, respectively).
Mego et al. [107]	To assess if probiotics can prevent diarrhea caused by irinotecan	Probiotics group (*n* = 23) Placebo group (*n* = 23)	Probiotic formula Colon Dophilus: *Bifidobacterium breve* HA‐129 *Bifidobacterium bifidum* HA‐132 HA *Bifidobacterium longum* HA‐135 *Lactobacillus rhamnosus* HA‐111 *Lactobacillus acidophilus* HA‐122 *Lactobacillus casei* HA‐108 *Lactobacillus plantarum* HA‐119 *Streptococcus thermopilus* HA‐110 *Lactobacillus brevis* HA‐112 *Bifidobacterium infantis* HA‐116	Metastatic colorectal cancer	Irinotecan	They found a lower incidence of diarrhea and enterocolitis when probiotics were administered instead of a placebo (0% vs. 8.7%, respectively). Patients in the probiotic group took lower doses of atropine, diphenoxylate, and loperamide than those in placebo group.
Mohebian et al. [124]	To assess the yogurt+ probiotic's efficacy for diarrhea associated with chemotherapy	Yogurt + probiotics: (*n* = 19) Yogurt: (*n* = 22) Control: (*n* = 25)	*Lactobacillus casei*, *Lactobacillus acidophilus*, *Lactobacillus rhamnosus*, *Lactobacillus bulgaricus*, *Bifidobacterium breve*, *Bifidobacterium longum*, *Streptococcus thermophiles*, fructooligosaccharide	Colorectal cancer	5‐fluorouracil	Yogurt with probiotics group had significantly fewer defecation, less severe diarrhea and better stool consistency than the control group (*p* < 0.05)
Motoori et al. [125]	To assess the impact of synbiotics on side effects in patients with esophageal cancer receiving neoadjuvant chemotherapy	Synbiotics group (*n* = 30) Control (Biofermin) group (*n* = 31)	Yakult BL Seichoyaku: *Bifidobacterium breve*, *Lactobacillus casei*, galacto‐oligosaccharides	Esophageal cancer	Docetaxel Cisplatin 5‐fluorouracil	In comparison to the control group, the frequency of severe diarrhea was significantly lower in the synbiotics group (*p* = 0.035).
Reyna‐Figueroa et al. [128]	To assess the contribution of probiotics to the gastrointestinal side effects of chemotherapy in Acute Leukemia patients	Probiotic group (*n* = 30) Control group (*n* = 30)	*Lactobacillus rhamnosus* GG	AML ALL	Prednisone orally Vincristine intravenously Daunorubicin intravenously l‐asparaginase intramuscularly	
Reyna‐Figueroa et al. [129]	To assess the impact of probiotics on complications related to post chemotherapy in children diagnosed with acute lymphoblastic leukemia	Probiotic group (*n* = 30) 2. Control group (*n* = 30)	*Lactobacillus rhamnosus* GG	ALL	Prednisone orally Vincristine intravenously Daunorubicin intravenously l‐asparaginase intramuscularly	When compared to the control group, the probiotic group experience a more than 60% decrease in diarrhea.

**TABLE 5:** Comparison of effective probiotic strains in animal and clinical studies.

Animal studies	Clinical studies
Selenium‐enriched *Bifidobacterium longum* DD98's	*Lactobacillus acidophilus* *Lactobacillus plantarum* *Lactobacillus casei* *Lactobacillus delbrueckii bulgaricus* *Bifidobacterium breve* *Bifidobacterium longum* *Bifidobacterium infantis* *Streptococcus salivarius thermophilus*
*Lactobacillus plantarum*, *Lactobacillus casei*, *Streptococcus faecalis* *Bifidobacterium brevis*	*Lactobacillus casei* *Lactobacillus acidophilus* *Lactobacillus rhamnosus* *Lactobacillus bulgaricus* *Bifidobacterium breve* *Bifidobacterium longum* *Streptococcus thermophilus*
*Akkermansia muciniphila BAA‐835*	*Bifidobacterium breve*
*Streptococcus thermophilus TH‐4*	*Bifidobacterium breve HA‐129*, *bifidum HA‐132*, *Bifidobacterium longum*
*Clostridium butyricum* *Bacillus mesentericus* *Streptococcus faecalis*	*Lactobacillus casei* *Lactobacillus acidophilus* *Lactobacillus rhamnosus* *Lactobacillus bulgaricus* *Bifidobacterium breve* *Bifidobacterium longum* *Streptococcus thermophiles*, *Fructooligosaccharide*
*Lactobacillus acidophilus* *Bifidobacterium bifidum*	*Bifidobacterium breve* *Lactobacillus casei galacto‐oligosaccharides*
*Lactobacillus rhamnosus 35*	*Lactobacillus rhamnosus GG*
*Streptococcus thermophilus TH‐4* *Bifidobacterium infantis*	*Bacillus licheniformis*
	*Lactobacillus acidophilus LAC‐361* *Bifidobacterium longum BB‐536*
	*Lactiplantibacillus plantarum HEAL9* *Lactiplantibacillus plantarum*

#### 2.4.1 Animal Model Studies

To alleviate diarrhea symptoms following chemotherapy, numerous microorganisms with different quantities, duration, and dosages were investigated in various animal models. In a number of investigations, it was discovered that therapy with a multi‐strain probiotic suspension significantly improved diarrhea in mice and rats receiving chemotherapeutic agents (Table 2).

Consistent with these findings, oral administration of 1 mL probiotic suspension of *Bifidobacterium brevis*, *Streptococcus faecalis*, *L. casei*, and *L. plantarum*, using a syringe for 7 days prior to the injection of chemotherapy drugs and then cyclically (5 days straight followed by a break of 5 days) until the animal's death, had a protective effect against diarrhea in male Wistar–Lewis rats with CRC under capecitabine treatment. In fact, no sign of diarrhea was found in rats with probiotic consumption. In addition, it seems that the antitumor activity of probiotics and their consumption as both prophylaxis and therapy and in a long and periodic manner were effective in improving tolerance to capecitabine [108]. Treatment with a 100 μL mixture of the probiotics comprising 1 × $10^{7}$ CFU of the *Lactobacillus acidophilus*, and *Bifidobacterium bifidum* (LaBi, Infloran, Italy) for 5 days, also demonstrated a significant reduction in diarrhea in both BALB/c [109] and CID/NOD mice, a more precise model of immunodeficiency in malignancy (*p* < 0.001 and *p* < 0.05, respectively) [110]. Based on these studies, rats receiving 5‐FU experienced diarrhea from Day 2 with a significant increase in Days 3 and 4. By Day 5, probiotics had considerably improved the diarrhea score compared to 5‐FU alone: from 2.00 to 1.42 ± 0.24 (*p* < 0.05) and 2.64 to 1.45(*p* < 0.001) in CID/NOD and Balb/c mice, respectively. Moreover, the effects of a suspension of probiotics containing rhamnosus variety of *L. casei* (Lcr35, Antibiophilus, France) were also investigated. The diarrhea score was dropped to 1.33 ± 0.17 (*p* < 0.05) and 0.80 (*p* < 0.001) in CID/NOD and Balb/c mice, respectively, after consumption of this probiotic. Although LaBi had a better effect than Lcr35 on reducing diarrhea in BALB/c mice, there was no difference between these two suspensions in SCID/NOD mice.

Previously, it was indicated that there is a link between high levels of TNF‐α and CID [111]. Consuming 0.5 mL of the probiotic BIO‐THREE (200 mg tablet) which contains the bacteria *Clostridium butyricum*, *Bacillus mesentericus*, and *S. faecalis* reduced TNF‐α levels in the intestine (*p* < 0.02) and, therefore, improved diarrhea symptoms in Kunming female mice receiving 30 mg/kg of the platinum‐based chemotherapy drug oxaliplatin [112].

In addition to different probiotic mixtures, new‐generation probiotics (NGPs) were also tested for their efficacy in ameliorating chemotherapy‐related side effects, especially diarrhea. NPGs are bacteria that are primarily obtained from commensal microbiota; they may also be genetically modified; however, there is little or no evidence that they can operate as health promoters [113]. Treatment with an NGP, *Akkermansia muciniphila* BAA‐835 ($10^{10}$ CFU), significantly reduced small intestine shortening (*p* = 0.03) and weight loss (*p* = 0.004) in female Balb/c mice undergoing 5‐FU therapy. Also, *A. muciniphila* BAA‐835 considerably lowered the symptoms of intestinal mucositis, alleviating the severity of the condition [114].

Since it was stated that *Bifidobacterium infantis* has a positive impact on digestive disorders, this probiotic was also evaluated in treatment of 5‐FU‐induced diarrhea in male Sprague–Dawley rats. Administration of 1 mL *B. infantis* both pre‐ and postoperative did not cause any diarrhea prior to 5‐FU injection and reduced diarrhea in rats taking chemotherapy (*p* < 0.05) [115]. *B. infantis* was found to lessen the severity of intestinal damage brought on by 5‐FU, limit weight loss (*p* < 0.05) and villous shortening (*p* < 0.05), and reduce the frequency of diarrhea (*p* < 0.05). NF‐kB is a crucial molecular player in 5‐FU‐induced injury to mucosa. Suppression of NF‐kB activity decreases 5‐FU‐induced inflammation and mucosal injury [116]. *B. infantis* is shown to be associated with decreased NF‐kB activity and cell destruction (*p* < 0.05), as more proliferating cells were seen in probiotic groups.

Moreover, the effect of probiotics on other chemotherapy drugs besides 5‐FU was also investigated. A novel probiotic, *S. thermophilus* TH‐4, also significantly improved gastrointestinal complications caused by doxorubicin in female Dark Agouti rats [117]. However, another study revealed that all methotrexate‐treated rats had significantly damaged jejunal (proximal) and ileal (distal) mucosa (*p* < 0.05) in comparison to saline and TH‐4 controls, and animals given MTX+TH‐4 did not differ from the MTX controls in any way [118].

Selenium has also been demonstrated to be an advantageous trace element in reducing chemotherapy‐induced toxicity [119]. Therefore, the preventive effects of *Se‐Bifidobacterium longum* DD98, an Se‐enriched probiotic, to reduce hepatic and intestinal toxicities caused by CPT‐11, was evaluated. Se‐enriched probiotics may offer stronger protection against toxicities of CPT‐11 than pure probiotic therapy, since *Se‐B. longum* DD98 showed a greater ability for protection than *B. longum* DD98. In an animal experiment, CPT‐11 produced considerable weight loss, debilitating diarrhea, and fecal occult blood, all of which were symptoms of marked intestinal toxicity (*p* < 0.05). When compared to Na_2_SeO_3_ and *B. longum* DD98, *Se‐B. longum* DD98 collectively had more effective soothing effects: lowering the rate of diarrhea to 50%, 75%, and 25%, respectively [120].

Studies conducted on various models of mice and rats revealed the therapeutic potential of probiotics in minimizing chemotherapy‐related side effects including mucositis, diarrhea, and weight loss. Generally, treatment with probiotic mixtures regulated the gut microbiota and pro‐inflammatory responses and, as a result, increased animal tolerance to chemotherapy agents including 5‐FU, methotrexate, and CPT‐11. Despite the positive response to most probiotics, investigations on *S. thermophilus* TH‐4 had contradictory results that required further investigations. In fact, there are very limited data on diarrhea brought on by chemotherapy apart from mucositis. Further animal studies aiming to investigate diarrhea and different strains of probiotics, doses, and duration are needed.

#### 2.4.2 Human Studies

##### 2.4.2.1 Effects of Probiotics on RID

Several studies assessed the effectiveness of probiotics and synbiotics in decreasing diarrhea following anti‐cancer treatments; however, the reported results are conflicting (Table 3).

Four different studies investigated the use of probiotics in RID. Ahrén et al. showed that the administration of probiotics containing *Lactiplantibacillus plantarum* (HEAL9) and *Lactiplantibacillus plantarum* 299 in patients undergoing pelvic radiotherapy at a minimum dose of 40 Gy had no significant effect on the mean daily number of loose stools. However, the mean number of days with at least one loose stool significantly decreased from 15.04 ± 8.92 days in the placebo group to 8.65 ± 5.93 days in the high‐dose probiotics group ($5\times 10^{10}$ colony‐forming units/capsule twice daily) (*p* = 0.014). The outcomes persisted for 14 days following the radiation [10]. Similarly, a clinical trial study followed up patients treated with pelvic radiation who consumed *Bifilact* probiotic (a combination of *L. acidophilus* LAC‐361 and *B. longum* BB‐536) at a concentration of 1.3 billion CFU, twice a day for the standard dose group or 10 billion CFU, three times a day for the high dose group. It demonstrated that although there was no significant difference in overall grade diarrhea incidence compared with the placebo group, at 60 days, the standard dose group had twice as many patients without moderate or severe diarrhea as the placebo group (*p* = 0.04) [121]. In another study on pelvic cancer patients treated with conventional radiotherapy to a total dose of 40–50 Gy, using *LactoCareO* probiotic (composed of *L. casei*, *L. acidophilus*, *Lactobacillus rhamnosus*, *Lactobacillus bulgaricus*, *Bifidobacterium breve*, *B. longum*, and *S. thermophilus*) were found to be beneficial. The findings revealed a reduction in the number of bowel movements per day (*p* = 0.003 and 0.006), the severity of the diarrhea (*p* = 0.001 and 0.001), and the need for antidiarrheal medicine (*p* = 0.021 and 0.041). Moreover, there was a change in the consistency of the stool in patients who received probiotics alone or in combination with honey (*p* = 0.004 and 0.005). However, the probiotic triggered some side effects, such as stomach pain and bloating [63]. Moreover, the study of Du et al. determined the protective effects of *Bacillus licheniformis* on craniospinal radiation‐induced toxicity in children with central nervous system tumors. A therapeutic dose of this probiotic was revealed to reduce gastrointestinal toxicities such as diarrhea by balancing the gut microbiota [122].

These evidences suggested that probiotics may help alleviate radiation‐induced diarrhea in some cases. However, since the evidence is limited and patients, toxicity scales, radiation regimens, and probiotics vary throughout studies, it is challenging to draw a conclusion with certainty. As a result, conducting additional RCTs is highly recommended to confirm the findings.

##### 2.4.2.2 Effects of Probiotics on CID

There are many chemotherapy drugs that cause diarrhea. The most prevalent drugs include topoisomerase I inhibitors (irinotecan, topotecan), fluoropyrimidines (e.g., 5‐FU and its prodrugs, capecitabine), and other drugs such as cisplatin, docetaxel, oxaliplatin, and cytarabine [123]. The role of probiotic consumption in alleviating diarrhea symptoms induced by these chemotherapy drugs is debatable.

Mohebian et al. investigated the effectiveness of yogurt with probiotics on 5‐FU‐related diarrhea. The probiotic capsule (containing 500 mg of *L. casei*, *L. acidophilus*, *L. rhamnosus*, *L. bulgaricus*, *B. breve*, *B. longum*, *Streptococcus thermophiles*, and *fructooligosaccharides*) given for a week to patients with CRC improved diarrhea symptoms, so that the yogurt with probiotics group had significantly fewer defecation, less severe diarrhea, and better stool consistency compared to the control group (*p* < 0.05) [124]. In another clinical trial, 61 esophageal cancer patients recieving docetaxel, cisplatin, and 5‐FU administered synbiotics containing *B. breve*, *L. casei*, and galacto‐oligosaccharides showed reduced diarrhea frequency (*p* = 0.035) [125]. The study by Mego et al. on individuals with CRC who received an injection of irinotecan revealed the same outcomes. They found a lower incidence of diarrhea and enterocolitis when probiotics were administered instead of a placebo (0% vs. 8.7%, respectively). In addition, patients who consumed probiotics reported using less loperamide and atropine to manage the symptoms of diarrhea [107]. These findings contrast with the results of a cohort research that investigated the prophylactic effects of oral VSL#3 probiotics on dacomitinib‐induced gastrointestinal toxicities in individuals diagnosed with non‐small cell lung cancer. The probiotic failed to lower the diarrhea burden index (DBI) (*p* = 0.860) and reduce the need for concomitant drug treatment (loperamide) when compared to the placebo [126].

Since pediatric and adult patient populations can respond to treatments differently, it is also essential to review the efficacy of probiotics on CID in children. Results from clinical trials conducted in pediatric patients are controversial. Eghbali et al. performed a study on 113 children with acute lymphocytic leukemia (ALL) to ascertain the role of LactoCare synbiotic administration in chemotherapy‐related gastrointestinal toxicity. They found that using $5\times 10^{9}$ CFU of LactoCare (composed of *L. casei*, *L. acidophilus*, *L. rhamnosus*, *L. bulgaricus*, *B. breve*, *B. longum*, *S. thermophilus*, and *prebiotic fructooligosaccharides*) for a week could reduce the severity and frequency of diarrhea. The findings demonstrated a reduction in the need for rescue medication (metoclopramide and loperamide) and the frequency of diarrhea in the patients who received LactoCare treatment, as they had a 1.45 times lower risk of developing diarrhea than the placebo group patients. (*p* = 0.048, odds ratio: 1.45; confidence interval: 1.17–4.01) [127].

Besides, another clinical trial study was carried out on 60 pediatric patients with acute leukemia who exhibit chemotherapy‐induced toxicities. Thirty of them were given 5 × 109 UFC *L. rhamnosus* GG. Probiotic (LrGG) twice a day for up to 7 days while receiving chemotherapy. The remaining 30 patients underwent the same chemotherapy regimen at the same time, but they were not administered probiotics. Following up on patients while they were in the hospital revealed that 10% of the control group's patients had diarrhea, whereas the probiotic group did not experience any cases of diarrhea. Then, the pediatric patients were followed for chemotherapy‐induced toxicities for 30 days after leaving the hospital. There were also statistically significant variations in the incidence of gastrointestinal symptoms between the probiotic and control groups (*p* = 0.009). In diarrhea, a reduction of over 60% was seen with the administration of probiotics compared to the non‐probiotic group. Therefore, the LrGG probiotic has been discovered to be risk‐free and successful in preventing the onset of diarrhea during hospitalization and post‐discharge follow‐up [128, 129]. Conversely, the study of Wada et al. determined that using *B. breve* probiotics in patients receiving chemotherapeutic agents for pediatric malignancies did not significantly reduce the onset and length of diarrhea, which might be induced by the incorrect dosage of probiotics [130].

Despite a limited amount of literature, the results are promising and mostly corroborate the idea that probiotics could be a safe, practical, and inexpensive method to shield both adult and pediatric cancer patients from chemotherapy‐induced diarrhea, which can deteriorate their condition and prolong hospitalizations. However, to determine their effectiveness, better‐designed studies are needed to evaluate cost‐effectiveness, hospital stay, morbidity, and mortality.

### 2.5 Probiotics' Safety and Cost

Extended hospital stays, electrolyte imbalances, circulatory failure, and other CID‐related conditions raise the cost of cancer therapy for patients. It is suggested that probiotics not only help to prevent CID but also lead to cost savings due to a lower risk of CID complications and reduced health‐care expenses. In addition, a comprehensive analysis of probiotics found that probiotics can reasonably reduce healthcare‐associated diarrhea in hospitalized adult patients, including antibiotic‐associated diarrhea (AAD) and *C. difficile*‐associated disease (CDAD) [131]. Moreover, a cost‐effectiveness analysis, conducted in a provincial health‐care system, revealed that the use of oral probiotics reduced the risk of CDAD from 5.5% to 2% while resulting in an average overall cost per patient treated of $327, as opposed to $845 per patient in the usual care strategy [132].

Although probiotics are beneficial, the negative effects and potential repercussions of long‐term use should not be underestimated. A recent study has reviewed the potential side effects of probiotics. The study found that some possible side effects of probiotics include infections such as bacteremia, gastrointestinal issues such as vomiting, nausea, spasms, diarrhea, bloating, thirst, and taste disturbance, as well as skin side effects such as skin rashes or mild acne. Moreover, the study has also noticed some side effects in cancer patients including endocarditis, sepsis, allergic sensitization, risk of autoimmune disorders, and chronic diseases [133].

However, there is limited research on the length and severity of side effects associated with probiotic intake. People with major illnesses or weakened immune systems are particularly susceptible to the harmful effects of probiotics. The safety of administrating probiotics in immunosuppressed and immunocompromised patients has been investigated. A systematic review of 57 clinical studies showed the safe administration of both pro‐ and synbiotics in immunocompromised patients [88]. These patients were of HIV‐infected, post‐surgical, autoimmune diseases, critically ill, and those who underwent organ transplantation. However, the authors believed there must be further detailed reports on adverse events to fully rely on these data. Anyhow, there were case reports of complications in immune‐deficient patients undergoing probiotic therapies, such as a *Saccharomyces cerevisiae* sepsis in an immune‐deficient infant upon probiotic therapy [89]. Probiotics have been shown to be effective and safe in HIV patients with little to no adverse event in several studies [90, 91, 92, 93]. In summary, probiotics have been shown to be safe in immunocompromised patients. However, it should be noted that safety profile of a certain strain cannot be extrapolate to other strains, and each disorder and strain should be studied separately.

As a result, the microbial medication should be precisely chosen using a series of important criteria, including as a thorough evaluation of specific human subpopulations to be treated, the medical indication under consideration, and the probiotic course of treatment. A study on Wistar–Lewis rats found that long‐term probiotic supplementation led to a systemic pro‐inflammatory response, including increased serum and tissue inflammatory cytokines, IL‐10 tissue levels, and an increase in the abundance of bacterial families linked to gastrointestinal inflammation. Also, long‐term probiotic medication dramatically increased cardiovascular risk factors, such as lipoprotein ratios in these rats [134]. Thus, choosing an appropriate probiotic mixture should be based on data from well‐designed trials regarding the probiotic's effectiveness during treatment. In addition, it is highly recommended to enhance our knowledge of the human gut microbiome and evaluate patients' health status before inoculating the gut with unidentified beneficial probiotics.

#### 2.5.1 Probiotic and Drug Interactions

Although probiotics are generally considered safe, their increasing popularity requires further investigation because of their possible effects on the safety, effectiveness, and metabolism of oral medications. Probiotics have the potential to affect the bioavailability of certain medications through several different mechanisms. These include changing the composition of the gut microbiota, affecting the expression of intestinal transporters, modifying the duration of intestinal transit or adherent mucous thickness, and altering local intestinal pH through the production of SCFAs. Antibiotics, antifungals, and other pharmaceuticals like prednisone, amoxicillin, and doxycycline are among those that have been reported to interact with probiotics [135].

Changes in the microbiome have been shown to have a significant effect on how the body reacts to chemotherapy drugs. Probiotics' ability to prevent cancer through a variety of pathways, such as bacterial translocation, gut homeostasis maintenance, immunomodulation, and overall antitumor effect, has also been thoroughly studied [136]. However, little is understood about how probiotics and anticancer medications interact kinetically. Irinotecan was found to have an interesting interaction with probiotics, suggesting that its effects may be either useful or detrimental according to the conditions of the local microbiota. During the breakdown of irinotecan, the stomach excretes SN‐38 G, which β‐glucuronidase subsequently partially deconjugates to replenish SN‐38. This reaches colon epithelial cells and might cause side effects such as neutropenia and diarrhea that could be lethal, decreasing the efficacy of treatment [137]. Wallace et al. found that β‐glucuronidase, produced by *E. coli*, *Bacteroides vulgatus*, and *Clostridium ramosum* in the intestinal lumen, increases the probability of adverse outcomes by converting SN‐38 G back to SN‐38. On the other hand, *Limosilactobacillus reuteri and B. infantis* did not affect irinotecan therapy because they do not have the β‐glucuronidase gene [138].

Furthermore, some evidence suggests that carefully controlling the gut flora with a suitable probiotic may accelerate the response to PD‐1 blockers; however, additional study is needed to substantiate this notion [139, 140]. Table 6 illustrates some examples of interactions between certain species and chemotherapeutic drugs, suggesting that probiotics may have an impact on improving the response to cancer treatments.

**TABLE 6:** Several instances of drug‐microbiome interactions in cancer treatment.

Medication	Bacteria	Interaction	Reference
5‐Fluorouracil (5‐FU)	*F. nucleatum*	Promote the development of resistance to chemotherapy in colorectal cancer by modifying the chemotherapeutic response and activating the autophagy pathway	Yu et al. [141]
	*Lactobacillus acidophilus* and *Lactobacillus casei*	Enhance the apoptosis‐induction capacity of the 5‐FU	Baldwin et al. [142]
Cyclophosphamide (CTX)	*Gram‐positive bacteria*	Generate a specific subset of “pathogenic” T helper 17 (pT(H)17) cells and memory T(H)1 immune responses	Viaud et al. [143]
Oxaliplatin	*Gram‐negative bacteria*	Modulate myeloid‐derived cell functions in the tumor microenvironment	Iida et al. [144]
Anti‐PD‐1/PD‐L1 mAbs	*Bifidobacterium*	Enhance NK cell functions	Rizvi et al. [145]
	*Bifidobacterium*	Augment dendritic cell function leading to enhanced CD8(+) T cell priming and accumulation in the tumor microenvironment	Sivan et al. [146]
	*Akkermansia muciniphila*	Restore the efficacy of PD‐1 blockade in an interleukin‐12‐dependent manner by increasing the recruitment of CCR9 + CXCR3 + CD4+ T lymphocytes into mouse tumor beds	Routy et al. [140]
	*Ruminococcaceae family*	Enhance systemic and anti‐tumor immune responses mediated by increased antigen presentation, and improved effector T cell function in the periphery and the tumor microenvironment	Gopalakrishnan et al. [147]
	*Bifidobacterium longum*, *Collinsella aerofaciens*, and *Enterococcus faecium*	Improve tumor control, augment T cell responses, and greater efficacy of anti‐PD‐L1 therapy	Matson et al. [148]
Anti‐CTLA‐4 mAbs	*Bacteroidetes*	Cause resistance to the development of checkpoint‐blockade‐induced colitis	Dubin et al. [149]
	*Bifidobacterium*	Modulate metabolic functions of Treg cells	Wang et al. [150]
	*B. fragilis*	Increase efficacy of CTLA‐4 blockade	Vétizou et al. [151]
Camptothecin	*E. coli*	*E. coli* deletions increase CPT efficacy in a passive way that does not require bacterial metabolism, for instance through nutritional support of nucleotides to the host	García‐González et al. [152]
	*Comamonas*	Decrease CPT efficacy in a passive way that does not require bacterial metabolism, for instance through nutritional support of nucleotides to the host	
Floxuridine	*E. coli*	Increase drug efficacy through active bacterial metabolism	García‐González et al. [152]
	*Comamonas*	Decrease drug efficacy through active bacterial metabolism	

## 3 Conclusions

During cancer treatment, radiation and chemotherapy‐induced diarrhea (RID and CID) is a common and frequently incapacitating side effect that can result in several complications. The use of probiotics—described as living microorganisms that might benefit the host—has been suggested and extensively researched as a possible treatment and prevention method for diarrhea brought on by chemotherapy. Probiotics can reverse both CID and RID progression by restoring gut microbiota imbalance, and cellular junction integrity, and reducing inflammation. In this narrative review, we investigated a handful of clinical trials and animal research regarding the efficacy of probiotics. Various strains of *Lactobacillus*, *Bifidobacterium*, and *Streptococcus* were studied and discussed. The results showed a significant and most successful treatment with probiotics in the treatment of CID and radiation‐induced diarrhea. According to the findings, probiotics are a major and highly effective treatment for CID and radiation‐induced diarrhea. Further study is recommended to identify the most effective and safe probiotic strains, as well as dose adjustments for the CID and radiation‐induced diarrhea. Attention must be paid in immune‐deficient patients due to possible risks and future studies must also address the issues. Even though the current evidence is not enough to decide, they are still very supporting of adequate response.

## 4 Future Studies

Since the included studies are heterogeneous in study design, patient groups, and probiotic strains, conducting further trials seems essential to reaching firm results. These trials should focus on standardizing probiotic strain types and dosages to recognize their possible advantages and disadvantages. In addition, prolonged follow‐up periods should be a feature of future research to evaluate the long‐term impact of probiotics on side effects like chemotherapy or radiotherapy‐induced diarrhea. Moreover, to comprehend the effect of probiotics on chemotherapy‐induced diarrhea, researchers should concentrate on conducting trials with homogeneous sample sizes. For example, patients should be similar in terms of cancer types and stages as well as chemotherapy regimens. Multi‐center trials with larger patient populations are also beneficial to fully assess the effectiveness of probiotics on diarrhea symptoms induced by chemotherapy drugs. Furthermore, mechanistic studies are required to clarify the pathways via which probiotics may lessen diarrhea brought on by chemotherapy or radiotherapy. This could help develop more focused therapies.

## Author Contributions

**Sanaz Khorashadizadeh:** conceptualization (equal), data curation (equal), investigation (equal), validation (equal), visualization (equal), writing – original draft (equal), writing – review and editing (equal). **Sara Abbasifar:** conceptualization (equal), data curation (equal), investigation (equal), validation (equal), visualization (equal), writing – original draft (equal), writing – review and editing (equal). **Mohammad Yousefi:** conceptualization (equal), data curation (equal), investigation (equal), validation (equal), visualization (equal), writing – original draft (equal), writing – review and editing (equal). **Farzad Fayedeh:** conceptualization (equal), data curation (equal), investigation (equal), project administration (equal), validation (equal), visualization (equal), writing – original draft (equal), writing – review and editing (equal). **AmirAli Moodi Ghalibaf:** conceptualization (equal), project administration (equal), supervision (equal), validation (equal), visualization (equal), writing – original draft (equal), writing – review and editing (equal).

## Ethics Statement

The authors have nothing to report.

## Consent

The authors have nothing to report.

## Conflicts of Interest

The authors declare no conflicts of interest.

## Data Availability

The data that support the findings of this study are available on request from the corresponding author. The data are not publicly available due to privacy or ethical restrictions.
